# Mutation of *Inositol 1,3,4-trisphosphate 5/6-kinase6* Impairs Plant Growth and Phytic Acid Synthesis in Rice

**DOI:** 10.3390/plants8050114

**Published:** 2019-04-29

**Authors:** Meng Jiang, Yang Liu, Yanhua Liu, Yuanyuan Tan, Jianzhong Huang, Qingyao Shu

**Affiliations:** 1 National Key Laboratory of Rice Biology, Institute of Crop Sciences, Zhejiang University, Hangzhou 310058, China; mengjiang@zju.edu.cn (M.J.); 21616041@zju.edu.cn (Y.L.); yanhual624@163.com (Y.L.); tanyy@zju.edu.cn (Y.T.); jzhuang@zju.edu.cn (J.H.); 2 Hubei Collaborative Innovation Center for Grain Industry, Yangtze University, Jingzhou 434025, China; 3 Institute of Nuclear Agricultural Sciences, Zhejiang University, Hangzhou 310058, China

**Keywords:** genome editing, growth, *ositpk6*, phytic acid, rice

## Abstract

Inositol 1,3,4-trisphosphate 5/6-kinase (ITPK) is encoded by six genes in rice (*OsITPK1-6*). A previous study had shown that nucleotide substitutions of *OsITPK6* could significantly lower the phytic acid content in rice grains. In the present study, the possibility of establishing a genome editing-based method for breeding low-phytic acid cultivars in rice was explored, in conjunction with the functional determination of OsITPK6. Four *OsITPK6* mutant lines were generated by targeted mutagenesis of the gene’s first exon using the CRISPR/Cas9 method, one (*ositpk6_1*) with a 6-bp in-frame deletion, and other three with frameshift mutations (*ositpk6_2*, _*3*, and _*4*). The frameshift mutations severely impaired plant growth and reproduction, while the effect of *ositpk6_1* was relatively limited. The mutant lines *ositpk6_1* and _*2* had significantly lower levels (−10.1% and −32.1%) of phytic acid and higher levels (4.12- and 5.18-fold) of inorganic phosphorus compared with the wild-type (WT) line. The line *ositpk6_1* also showed less tolerance to osmotic stresses. Our research demonstrates that mutations of *OsITPK6*, while effectively reducing phytic acid biosynthesis in rice grain, could significantly impair plant growth and reproduction.

## 1. Introduction

Myo-inositol-1,2,3,4,5,6-hexakisphosphate (IP_6_), also known as phytic acid (PA), is the main storage form of phosphorous (P) (65–80%) in cereal and legume seeds, accounting for ~1.5% of the dry weigh [1]. In most cereal grains, PA exists as mixed salts (phytates) in protein storage bodies and can chelate several mineral cations, including Zn^2+^, Fe^2+^, Ca^2+^, and Mg^2+^ [2]. During seed germination, endogenous grain phytase is activated to degrade phytate, releasing myo-inositol, phosphorus, and bound mineral cations [3], which are utilized by the developing seedlings. The PA biosynthetic pathway is still not well defined, but a number of genes involved in its biosynthesis or transport have already been cloned in several plants. Mutations of these genes could result in low-phytic-acid (*lpa*) grains in rice [4,5,6,7,8,9,10,11,12,13,14] and other plants, e.g., wheat [15] and maize [3,16,17]. In rice, 12 genes have been identified that catalyze the production of intermediate inositol polyphosphates in seeds [18].

Inositol 1,3,4-trisphosphate 5/6-kinase (ITPK) plays a pivotal role in phytic acid biosynthesis, whereby the inositol triphosphate (IP_3_) molecule is further phosphorylated at the 5th or 6th position [19,20]. ITPK belongs to the ATP-grasp fold proteins group [21] and is conserved from plants to humans with diverse functions. ITPK has even been found in the anaerobic protozoan *Entamoeba histolytica* [22], where its transcription is slightly induced by heat shock, demonstrating its role in the cellular response to stress [21]. The first plant ITPK, AtITPK1, was identified in *Arabidopsis* [20]. AtITPK1 is involved in photomorphogenesis possibly by interacting with the constitutive photomorphogenic (COP) signalosome under red light [23]. The kinase activity of AtITPK1 is indispensable for maintaining inorganic phosphorus (Pi) homeostasis under Pi-replete conditions, and *itpk1* mutants exhibited decreased levels of IP_6_ and diphosphoinositolpentakisphosphate (IP_7_). Disruption of another ITPK family enzyme, ITPK4, also caused depletion of IP_6_ and IP_7_ but did not display similar Pi-related phenotypes as *itpk1* [24]. AtITPK4 is an outlier to its family and does not display inositol 3,4,5,6 tetrakisphosphate 1-kinase activity; rather, it displays inositol 1,4,5,6-tetrakisphosphate and inositol 1,3,4,5-tetrakisphosphate isomerase activity [21]. AtITPK2 was required for seed coat development and lipid polyester barrier formation [25], and ABA or phosphorus deficiency could induce *AtITPK2* expression. In maize (*Zea mays* L.), ZmITPK1 exhibits multiple inositol phosphate kinase activities and is involved in phytic acid biosynthesis in developing seeds [17]. In soybean (*Glycine max* L.), GmITPK1 is a potential candidate for developing low-phytate soybean [26], and GmITPK2 may play a role as a dehydration and salinity stress regulator [27].

In rice (*Oryza sativa* L.), the *OsITPK* genes can be divided into three sub-families [18]. *OsITPK1*, *OsITPK2*, and *OsITPK3* belong to subgroup I, each with 10 exons and 9 introns; *OsITPK4* and *OsITPK5* belong to subgroup II, which has no intron; and *OsITPK6* belongs to subgroup III, with 12 exons and 11 introns. OsITPK2 is a negative regulator of osmotic stress signaling [28], and its disruption could affect the expression of some of its homologous genes, *OsITPK1* and *OsITPK4* [29]. The expression of *OsITPK4*, but not of *OsITPK1, 2, 3*, and *5* can be strongly induced by cold and heat stresses [29]. The IP_3_ level was not affected by the *ositpk2* mutation, probably owing to redundant functions of other homologs [29].The expression of *OsITPK6* could also be induced by heat [29], and mutations of *OsITPK6* were already demonstrated to result in significant reduction of IP_6_ in rice grains [30], i.e., mutant lines with the amino acid substitution P522L had IP_6_ content about half that of the wild-type (WT) line. Among the *ositpk6* mutants, one line with a P522L amino acid substitution had agronomic performance (seed weight, germination, and seedling growth) similar to that of its WT parent, suggesting *OsITPK6* could be a desirable target of mutagenesis for breeding yield-competitive *lpa* rice [30]. Since the binding site for nucleotide or ATP is between 200 and 500 amino acids in OsITPK6, the substitution mutation (P522L) is localized outside of this binding region. The effect of the P522L mutation in OsITPK6 on IP_6_ biosynthesis could be related to the interaction of the enzyme with another substrate inositol polyphosphate. On the other hand, a splicing mutant of *OsITPK6* at the 9th intron showed a more severe *lpa* phenotype: lower phytic acid content with reduced seed set [30]. Hence, it would be worthwhile to examine the function of OsITPK6 by generating more and different mutants, particularly by disruption of the ATP-binding region.

The clustered regularly interspaced short palindromic repeats (CRISPR) and CRISPR-associated protein 9 (Cas9) system, CRISPR/Cas9, is an efficient and precise genome-editing technique and has the potential to be used for crop improvement [31,32,33], including rice [34,35,36]. In the present study, we explored the possibility of establishing a genome-editing-based method for the fast breeding of yield-competitive *lpa* rice by evaluating *OsITPK6* mutants generated by CRISPR/Cas9-mediated mutagenesis. Our results showed that mutation of *OsITPK6* not only significantly reduced the accumulation of IP_6_ in rice grains but also impaired plant growth and tolerance to abiotic stress.

## 2. Results

### 2.1. Mutations of OsITPK6 and Development of Homozygous Transgene-Free Mutant Lines

**Figure 1:**
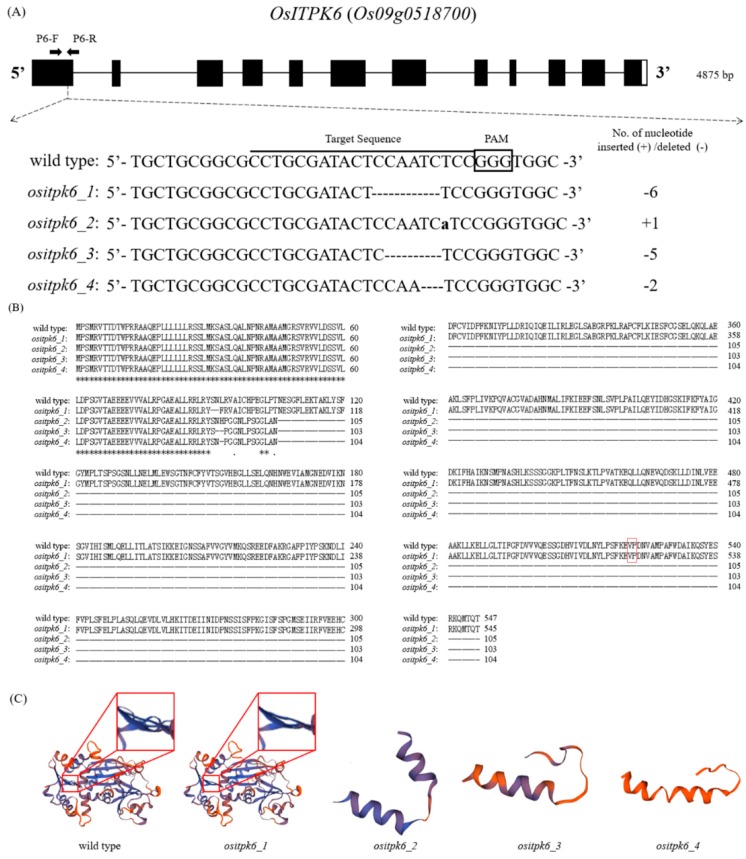
Schematic diagram of *OsITPK6* and sgRNA target site for CRISPR/Cas9-mediated mutagenesis of *OsITPK6*, and prediction of the related wild-type and mutant proteins. (**A**) Exons, introns, and UTRs are indicated by solid boxes, lines, and blank boxes, respectively. P6-F and P6-R are primers for mutation genotyping, and their positions are indicated by arrowheads. Mutation identified within the target site of *OsITPK6* generated through CRISPR/Cas9-mediated genome editing in rice. The PAM sequences (NGG) are boxed, and the 20-nt target sequences are underlined. Mutations are shown in lowercase letters (for insertions) or ‘–’ (for deletions). (**B**) The amino acid sequences of mutant proteins were aligned to that of the wild-type protein using the Clustal Omega Multiple Sequence Alignment (https://www.ebi.ac.uk/Tools/msa/clustalo/). The numbers represent the total number of amino acids, and the amino acid V521 and P522 are highlighted in red box. (**C**) The three-dimensional structures of OsITPK6 and its mutants were analyzed on SWISS-MODEL (https://www.swissmodel.expasy.org/).

In total, we obtained 23 hygromycin phosphotransferase (HPT)-positive T_0_ plants transformed with the CRISPR/Cas9 vector pH-itpk6. Among them, seven plants were found mutated at the target region, which represents an editing efficiency of 30.4%. T_1_ plants were tested for the presence of mutations and T-DNA, and transgene-free T_1_ plants with four types of mutation were identified. The mutations included a single-nucleotide (nt) insertion and three types of deletion (Figure 1A). Seeds were harvested from T_1_ plants with different mutations and developed into homozygous mutant lines, which were designated *ositpk6_1, _2*, *_3*, and *_4*.

The *ositpk6_1* (a 6-nt in-frame deletion) mutation would result in the loss of two amino acids at positions 91 to 92 (Figure 1B). In contrast, all the other three mutations, i.e., *ositpk6_2* (a 1-nt insertion), *ositpk6_3* (a 5-nt deletion), and *ositpk6_4* (a 2-nt deletion), would generate a premature stop codon almost right after the mutation site and, hence, significantly shorten the encoded proteins (Figure 1B). Consequently, the *ositpk6_2*, *_3* and *_4* mutant alleles were predicted to produce proteins of only 105, 103, and 104 amino acids, respectively (Figure 1C). Analysis of ITPK6 proteins of six organisms indicated that the two amino acids missing in the *ositpk6_1* mutant were located in a highly conserved segment (Figure S1), suggesting the mutation of *ositpk6_1* could have a potential functional consequence.

### 2.2. Impact of ositpk6 Mutations on Plant Growth and Seed Germination

**Figure 2:**
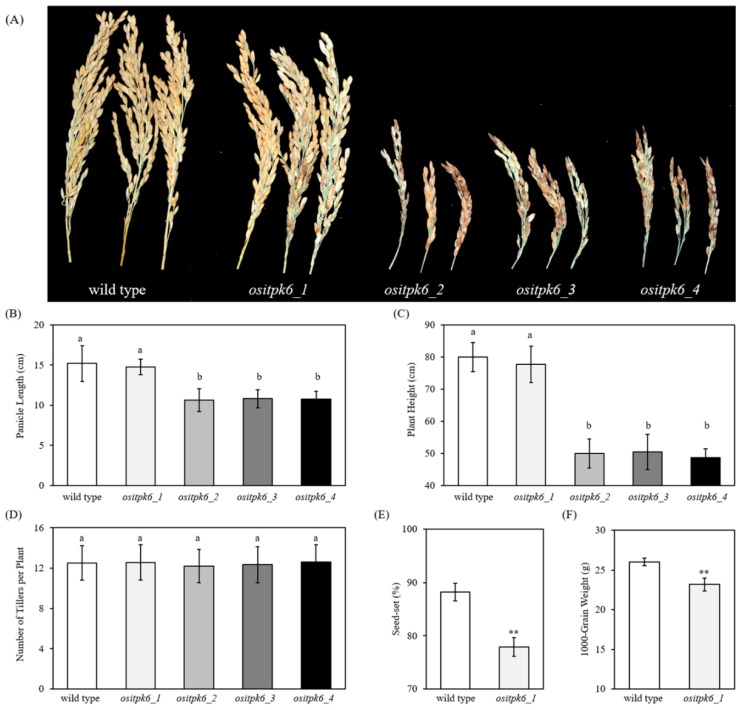
Agronomic traits of mutant *OsITPK6* line and wild-type plant. (**A**) Panicle phenotype of mutant *OsITPK6* and wild-type plants. (**B**–**D**) Twenty replicates were performed for four *OsITPK6* mutant lines and the wild-type plant. Error bars represent the standard error. The different letters show significant differences at a probability of *p* ˂ 0.05. (**E**,**F**) Twenty replicates were performed for four *OsITPK6* mutant lines and the wild type. Error bars represent the standard error. Data with an asterisk(s) are significantly different from those of the wild type (* *p* < 0.05, ** *p* < 0.01).

**Figure 3:**
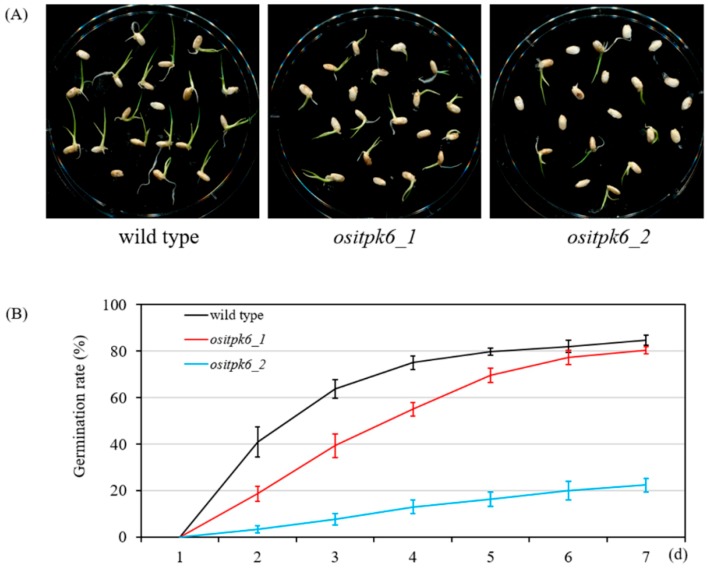
Germination rate of mutant seeds. (**A**) The pictures were taken on the 5th day after soaking. (**B**) The germination rate was recorded from 1 to 7 days after soaking, and three replicates were examined in each group.

Plant growth of *ositpk6_2*, *_3*, and *_4* was significantly impaired. First, their panicles were significantly shorter (−30.1%, −28.8%, and −29.1%, respectively) than that of the WT parental cultivar Xidao 1 (Figure 2A). Second, the mutant panicles had a high percentage of empty grains with darkened glumes (Figure 2B). Third, the height of the mutant plants was significantly reduced (−37.5%, −36.9%, and −39.1%, respectively) compared with that of their parental cultivar Xidao 1 (Figure 2C). The impact of *ositpk6_1* on plant growth and seed set was limited and not obvious (Figure 2A–C). No significant differences of tiller number per plant were observed between Xidao 1 and all four *ositpk6* mutant lines (Figure 2D). Compared with Xidao 1, the seed set and 1000-grain weight of *ositpk6_1* were also significantly decreased (−11.7% and −10.8%, respectively) (Figure 2E,F). Due to the extremely low seed set, we were not able to harvest enough seeds from *ositpk6_3* and *ositpk6_4* for the evaluation of 1000-grain weight and other characteristics. The germination of *ositpk6_1* was slower in the first three days (Figure 3A) but gradually caught up with that of the WT after five days and reached ~80% on the 7th day (Figure 3B). The germination rate of *ositpk6_2* was far lower than that of the WT (Figure 3A), being only ~20% on the 7th day (Figure 3B).

### 2.3. Effect of ositpk6 Mutations on Inorganic Phosphorus (Pi), Phytic Acid Phosphorus (PA-P), and Total Phosphorus (TP) in Brown Rice

**Figure 4:**
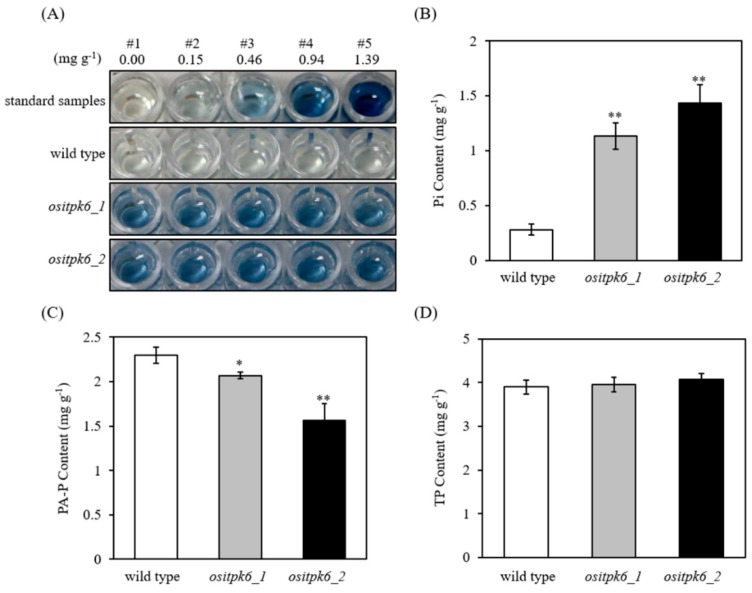
Inorganic P (Pi), phytic acid P (PA-P), and total phosphorus (TP) contents of the mutant *OsITPK6* lines and the wild type. (**A**) Qualitative assay of inorganic phosphorus (Pi) in mutant seeds. The concentration of the Pi standard samples is shown above. Five replicates were performed for two *OsITPK6* mutant lines and the wild type. (**B**–**D**) Six replicates were performed for two *OsITPK6* mutant lines and the wild type. Error bars represent the standard error. Data with an asterisk(s) are significantly different with respect to the wild-type data (* *p* < 0.05, ** *p* < 0.01).

A colorimetric assay showed that *ositpk6_1* and *ositpk6_2* had significantly higher Pi levels than the control (Figure 4A). To quantify the mutational effect of *ositpk6_1* and *ositpk6_2,* the Pi, PA-P, and TP contents were assessed in seeds of these two mutant lines, together with Xidao 1 as the WT control. All mutant lines had significantly lower levels of PA-P and higher levels of Pi compared with the control, while TP was not significantly different from that of the control (Figure 4B–D). *ositpk6_1* and *ositpk6_2* had Pi levels of 1.13 mg g^−1^ and 1.43 mg g^−1^, respectively, which were 4.12- and 5.18-fold higher than those of the control (0.28 mg g^−1^), respectively (Figure 4B). Xidao 1 seeds had a PA-P content of 2.30 mg g^−1^, which was significantly greater than those of the two mutant lines; the reduction of PA-P levels was 10.1% and 32.1% in *ositpk6_1* and *ositpk6_2*, respectively (Figure 4C). Xidao 1 seeds had a TP content of 3.9 mg g^−1^, which was not significantly different from those of the two mutant lines (Figure 4D).

### 2.4. Effect of ositpk6 Mutation on Stress Tolerance

**Figure 5:**
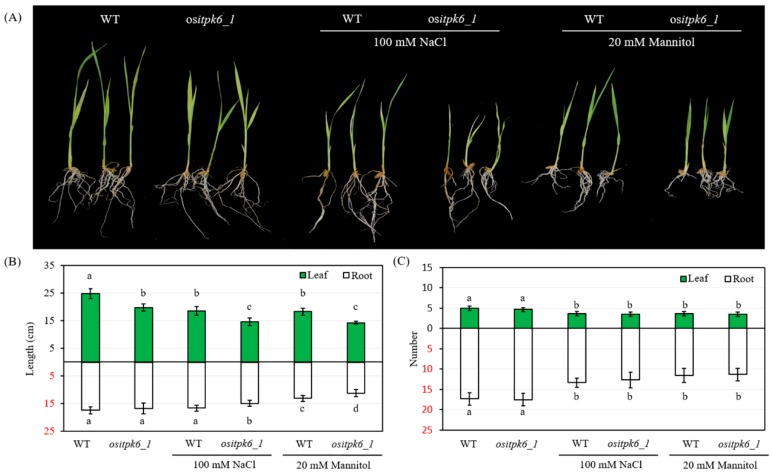
The phenotypes of the mutant *OsITPK6_1* and wild type under salt stress (100 mM NaCl) and drought stress (20 mM mannitol). (**A**) The picture was taken on the 7th day after treatment. (**B**,**C**) Six replicates were performed for the *OsITPK6* mutant line and the wild type. Error bars represent the standard error. The different letters show significant differences at a probability of *p* < 0.05.

To further test whether the mutation also had any impact on stress tolerance, we subjected the *ositpk6_1* and Xidao 1 plants to osmotic stress treatment (because of the limited number of seeds harvested and the low germination rate, *ositpk6_2*, was not further analyzed). The growth of *ositpk6_1* seedlings appeared to be inferior to that of the WT control grown either under normal or stressed conditions (Figure 5A). The shoot length of *ositpk6_1* was shorter than that of the WT with or without stress treatment (100 mM NaCl or 20 mM mannitol), while the root length of *ositpk6_1* was shorter than that of the WT only under stress (Figure 5A,B). There was no significant difference in the number of leaves and roots with or without stress treatment between *ositpk6_1* and WT (Figure 5C).

## 3. Discussion

ITPK6 is a unique gene in the ITPK gene family, and knowledge of its function has so far been very limited. The identification and characterization in rice of two *itpk6* mutant lines were reported in a study, which is the only study on the function of ITPK6 in all organisms [30]. Our present study demonstrated that the knockout of *OsITPK6* could severely impair plant growth and reproduction, implying that ITPK6 may play important roles in plant growth and development, in addition to the biosynthesis of inositol polyphosphates.

First, we observed that the *OsIPTK6* knockout mutants (*ositpk6_2*, *_3*, and *_4*) generated in the present study grew poorly, e.g., their plant height was reduced to almost half of that of the WT, and their fertility was almost abolished (Figure 2). These results suggested that *ITPK6* plays an important role not only in the vegetative growth but also in the reproduction of rice. Because the reduction of phytic acid content in *ositpk6_1* grains (−32.1%) was less than in the P522L mutant line (−46%) [30], the reduction of phytic acid alone could not explain the inferior performance of *ositpk6_1*. Further studies are needed to uncover the biological basis leading to the discrepancy between our present study and that reported in reference [30] regarding the mutational effect on rice growth and reproduction.

In the present study, we identified an *ositpk6* mutant, i.e., *ositpk6_1*, with a 6-bp deletion. Though only two amino acids are expected to be removed from the derived protein, *ositpk6_1* did reduce IP_6_ content. This may be because these two amino acids are located in a conserved region (Figure S1). We previously also observed a similar case of *lpa* rice, where a 6-bp deletion (and, hence, a deletion of two amino acids) in *OsSultr3;3* significantly reduced grain phytic acid content [14]. Although this mutation only reduced phytic acid content by less than 20%, it did exert a negative effect on seed set, grain weight, seed germination, and tolerance to abiotic stresses. This was somehow unexpected, because the P522L mutant line with a 46% reduction of phytic acid content still had normal plant growth as its WT parent [30]. Further studies are needed to fully evaluate the mutational effect by examining more *ositpk6* mutants.

The usefulness of CRISPR/Cas9-based mutagenesis for improving a particular trait is strictly dependent on the performance of the generated mutants. There are often trade-offs for mutating a gene for a specific purpose, and the overall performance of the mutated plant could be affected as a consequence of pleiotropic effects. Enlightened by the findings of Kim and Tai [30], we hoped to establish a fast and effective method for breeding yield-competitive, *lpa* rice cultivars by using genome-editing techniques. However, our results suggest that *OsITPK6* or its product plays an important role in multiple cellular processes, and simply knocking out *OsIPTK6* would impair rice growth and reproduction and, hence, would not work for our purpose.

Because [30] of the success in the production of *lpa* mutants without a significant negative impact on plant growth and seed development, it is still possible to generate *ositpk6* mutants without a significant effect on plant growth, if more appropriate vectors can be designed and more mutants are identified and assessed.

In summary, the present study demonstrates that the *OsITPK6* gene is essential for rice growth and reproduction.

## 4. Materials and Methods

### 4.1. CRISPR/Cas9 Vector Construction and Rice Transformation

To generate *OsITPK6* mutants, the 1st exon of *OsITPK6* (*Os09g0518700*) was chosen as a target (Figure 1A). The sgRNAs were designed by searching UniProt for precise positions (http://www.uniprot.org/), and CRISPR-P program (http://cbi.hzau.edu.cn/cgi-bin/CRISPR/) was used to minimize off-target effects [37]. Because of the homology of the target sequence among *OsITPK* genes, it is unlikely to cause mutations in the other five *OsITPK* homolog genes (Figure S2). DNA oligonucleotides were synthesized (Tsingke, Hangzhou, China) for the construction of a CRISPR/Cas9 vector, pH_itpk6, using the pHun4c12s as the starting plasmid, which harbors a *CYP81A6*-hpRNAi element [38] and was modified from pHun4c12 [39]. Correspondingly, the pH_itpk6 plasmid was transformed into *Agrobacterium tumefaciens* and used for rice transformation.

Rice calli were induced from mature seeds of the cultivar ‘Xidao 1’ (*O. sativa* L. *japonica*) and were transformed with the pH-itpk6 vector by *Agrobacterium*-mediated transformation according to reference [40]. Transgenic plantlets were regenerated from hygromycin-resistant calli and acclimatized inside a moist growth chamber (28 °C with a 12 h photoperiod) for one week before being transplanted to experimental facilities.

Ethics Approval and Consent to Participate: The experiments did not involve endangered or protected species. No specific permits were required for these locations/activities.

### 4.2. Mutation Detection in T_0_ Plants

For detection of transgenes and mutations in regenerated T_0_ plants, total genomic DNA was extracted from leaf tissues following a modified cetyltrimethylammonium bromide (CTAB) method [41]. The presence of the *HPT* gene was assessed by PCR using the primers HygR-F (5′-AGAAGAAGATGTTGGCGACCT-3′) and HygR-R (5′-GTCCTGCGGGTAAATAGCT-3′) [42]. Site-specific mutations were detected by PCR amplification using primer pairs flanking the designated target sites in *OsITPK6*, i.e., P6-F (5′-CTCGACCCATCCGGTGTTAC-3′) and P6-R (5′-AAATCGCAGGGGAGAGATCG-3′) (Figure 1A). The following generalized PCR program was used: 5 min at 94 °C, followed by 35 cycles of 30 s at 94 °C, 1 min at 60 °C, and 1 min at 72 °C, with a final extension of 10 min at 72 °C.

The PCR products were first subjected to HRM analysis for mutations according to reference [43], putative mutants were sequenced (TSINGKE, Hangzhou, China), and mutated sequences were decoded using the DSDecode program (http://skl.scau.edu.cn/dsdecode/) [44]. Mutations of a few selected plants were further confirmed by clone sequencing.

### 4.3. Development of Transgene-Free Mutant Lines

To obtain homozygous transgene-free mutants, T_1_ seedlings were foliar-sprayed with 1000 mg/L bentazon (approximately 100 mL/m^2^) at about the four-leaf stage according to a previous study [38]. At least five surviving T_1_ plants from each independent T_0_ plants were selected for further analysis of the presence of T-DNA and site-specific mutations. Eventually, four *OsITPK6* mutant lines (*ositpk6_1*, *_2*, *_3*, and *_4*) (Figure 1A) were identified; the T_2_ and advanced-generation seeds were used for the experiments.

### 4.4. Agronomic Traits Assay

The transgene-free mutant lines and their wild-type parent control (Xidao 1) were grown at the Experimental Farm of Zhejiang Zhijiang Seed Tec. Ltd., Hangzhou, China, during the summer season, and their agronomic traits were evaluated either in fields or post-harvest. Plants were grown in randomized plots, each with 60 plants. Twenty inner plants of each plot were evaluated for each parameter.

### 4.5. Seed Germination Assay

A controlled germination test was performed to assess seed germination capability, each with 100 seeds and replicated for three times [45]. Seeds were soaked in water for 48 h at 30 °C and then germinated on filter paper soaked with distilled water at 30 °C in the dark for one week, and the germination percentage was recorded daily.

### 4.6. Seed Phosphorus Assay

Seed inorganic P (Pi) levels were assayed qualitatively according to the micro-determination method developed in reference [46] with modifications. The qualitative assay was used for the identification of the high-inorganic-P (HIP) phenotype. The seeds were transferred to 96-well plates and extracted in 0.4 M HCl solution (10 μL per mg sample) overnight at 4 °C. Aliquots of 10 μL of supernatant per each sample were used for Pi level determination, according to reference [4] with slight modifications [6]. The development of a blue color implied increased level of Pi (HIP), while colorless samples typified the WT levels of parent varieties (Figure S1).

Seed Pi levels were also quantitatively determined according to reference [47] in triplicates, as follows. Brown rice grains were ground into rice flour, and ~400 mg rice flour per sample were extracted in 12.5% (*w*/*v*) TCA (trichloroacetic acid containing 25 mM MgCl_2_) by gentle shaking overnight at 4 °C. After centrifugation at 15,000× *g* for 10 min, the supernatants were used for Pi assay according to reference [2].

The PA content was determined for brown rice flour according to reference [48] by using a commercial assay kit (Megazyme, Irland) in triplicate. Briefly, ~1 g of brown rice grains was mixed with 20 mL of hydrochloric acid (0.66 M), stirred vigorously for 3 h at room temperature, and then centrifuged at 13,000 rpm for 10 min. Then, 0.5 mL of the supernatant was immediately neutralized by the addition of 0.5 mL of sodium hydroxide solution (0.75 M). The neutralized sample extract was subjected to an enzymatic dephosphorylation reaction procedure. The absorbances at 655 nm was determined using an ultraviolet spectrophotometer.

Total seed phosphorus and mineral elements were determined according to reference [49] in triplicate. Briefly, brown rice samples were digested in a microwave digestion system (Mars6, USA) at 160 °C for 40 min, using ~200 mg of sample in 7 mL of HNO_3_. The digested solution was concentrated at 140 °C for 2 h until less than 1 mL of solution was left and then brought to 30 mL with ultrapure water [50]. Total seed phosphorus and mineral elements were analyzed by Inductively Coupled Plasma–Mass Spectrometry (ICP–MS) (PerkinElmer, MA, USA).

### 4.7. Stress Treatment

For stress treatment, 14-day-old seedlings were planted in 1× Murashige and Skoog (MS) liquid medium [51], supplemented with 100 mM NaCl or 20 mM mannitol and grown for 7 days before sampling at midday.

### 4.8. Statistical Analysis

All statistical analyses were performed using the student’s *t*-test. The experimental data are presented with the mean standard errors (SE) based on three to six replications. The means were compared by ANOVA, and the significance of the differences between group means were calculated by the Bonferroni Post-tests.

## Supplementary Materials

The following are available online at https://www.mdpi.com/2223-7747/8/5/114/s1. Figure S1: Multiple sequence alignment of ITPK6s. All the data are from RAP-DB (http://rapdb.dna.affrc.go.jp/) and Gramene (http://www.gramene.org/). Analysis of ITPK6 sequences from six organisms using BioEdit software. Identical amino acid residues are boxed in same color. Short lines indicate gaps introduced during alignment. The mutant site of OsITPK6 is marked by a redbox. Figure S2. Multiple sequence alignment of *OsITPKs*. All data are from RAP-DB (http://rapdb.dna.affrc.go.jp/) and Gramene (http://www.gramene.org/). Analysis of *OsITPK1*, *OsITPK2*, *OsITPK3*, *OsITPK4*, *OsITPK5*, and *OsITPK6* sequences using the BioEdit software. Identical DNA bases are in the same color. Dotted lines indicate gaps introduced during alignment. The target site of *OsITPK6* is marked by a red box.
