# Supplementary figures and images for: Mutation of Inositol 1,3,4-trisphosphate 5/6-kinase6 Impairs Plant Growth and Phytic Acid Synthesis in Rice
Source: Plants (Basel). 2019 Apr 29;8(5):114. doi: 10.3390/plants8050114 (PMC6572258; doi:10.3390/plants8050114)

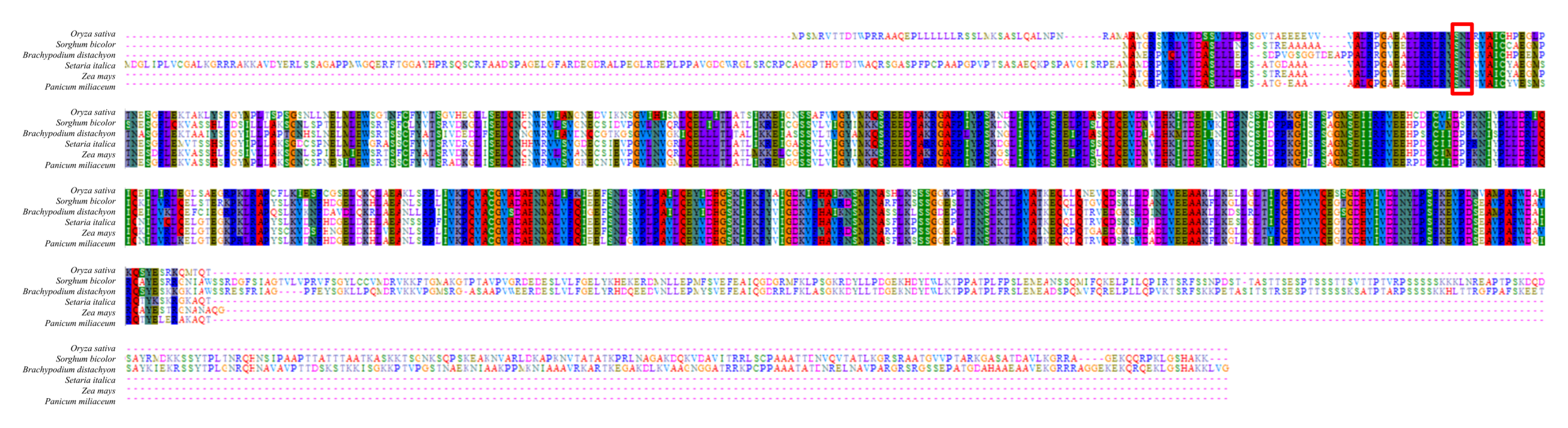

Supplement: Supplementary file 1 [file plants-08-00114-s001.zip › Supplementary File/Figure S1.tif]

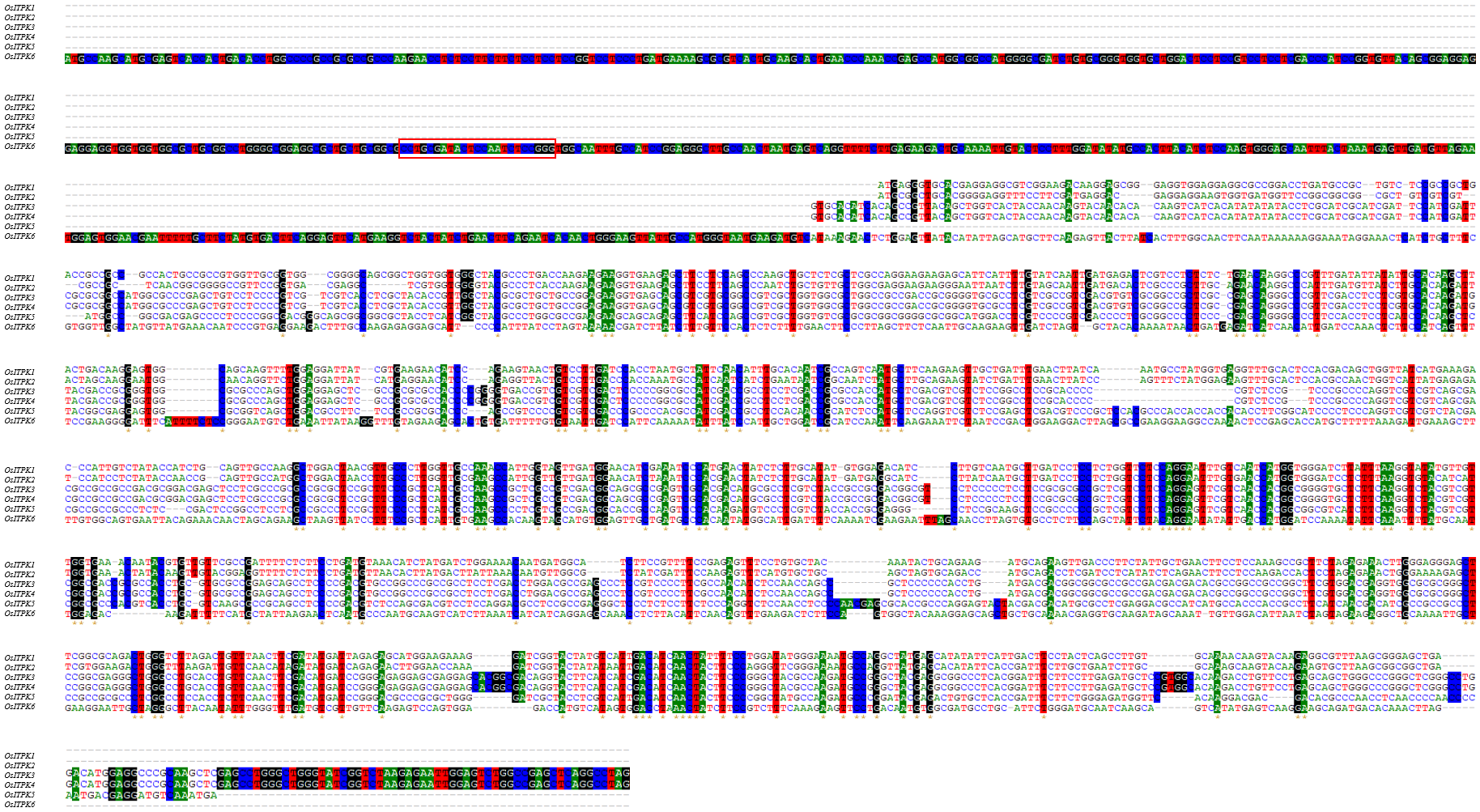

Supplement: Supplementary file 1 [file plants-08-00114-s001.zip › Supplementary File/Figure S2.tiff]
